# Precision therapeutic landscape for breast cancer: where are we headed?

**DOI:** 10.1016/j.lanwpc.2024.101024

**Published:** 2024-01-31

**Authors:** The Lancet Regional Health – Western Pacific

Breast cancer is the most commonly diagnosed cancer worldwide and caused 685,000 deaths globally in 2020. In Asia, breast cancer causes more than 300,000 deaths and is ranked first in 46 of 49 Asian countries in terms of incident cases in females in 2019. Advances in breast cancer therapeutics are needed to counteract the burden. Precision treatment, a strategy to tailor targeted therapy in a specific group of patients by considering individual variability in genes, environment, and lifestyle, might revolutionise the therapeutic landscape of breast cancer.

Development of treatment strategies for breast cancer depends on the progress of our understanding of the disease. Initially, breast cancer was thought to be a locoregional disease and surgical resection was the main treatment. Breast cancer was gradually recognised as a systemic disease when systemic chemotherapy led to improved patient outcomes, but the one-size-fits-all systemic therapy soon showed its limitations. With the advancement of molecular subtyping in breast cancer, subtype-based therapy has become the current standard treatment strategy. Nowadays, a plethora of novel trials and innovative technologies, such as omics assays, are shaping its therapeutic landscape to be more precise.

The current therapeutic scenario for breast cancer relies on the classification of three key subtypes based on hormone receptor (HR), including oestrogen receptor and progesterone receptor, and human epidermal growth factor 2 (HER2) status: HR-positive, HER2-positive, and triple-negative breast cancer (TNBC), in which cells are negative for all the three receptors. For early HR-positive breast cancer, endocrine therapy targeting the oestrogen and progesterone receptors is the mainstay strategy. But efficacy varies among patients due to the large heterogeneity, and the high risk of recurrence in the long term remains a crucial challenge. Hence, combination therapies in addition to chemotherapies or targeted agents are being tested. In the latest 4 year data from the monarchE trial, a combination of cyclin-dependent kinase 4 and 6 (CDK4/6) inhibitor abemaciclib, showed promising results, improving invasive disease-free survival by 6.4% while reducing the risk of recurrence. Nevertheless, along with the expanding use of CDK4/6 inhibitors in first-line metastatic cancer treatment and the adjuvant treatment of high-risk early-stage breast cancer, it should be noted that we are facing an increasing number of patients who do not respond to CDK4/6 inhibitor therapy and there are currently no standard treatment recommendations in international guidelines under this scenario.

Due to the development of multiple anti-HER2 antibodies, there is hope that early HER2-positive breast cancer is on the road to a cure. However, HER2-low breast cancer, which was recently found to account for about 55% of total breast cancer, has been more neglected in clinical practice. HER2-low breast cancer has traditionally been considered as HER2-negative but it had low level of HER2 immunohistochemistry, and traditional anti-HER2 therapies have poor efficacy in these patients. The hope relies on antibody-drug conjugates (ADCs), which comprises a monoclonal antibody tethered to a cytotoxic drug via a linker to deliver antibody to a targeted site. Unlike many other approved HER2-targeted therapies, trastuzumab deruxtecan, an anti-HER2 ADC has been found to effectively target tumour cells that express low levels of HER2. In 2022, DESTINY-Breast04, the first successful phase 3 clinical trial for the treatment of advanced HER2-low breast cancer, reported positive results for progression-free survival (PFS) and overall survival of trastuzumab deruxtecan, bringing a revolutionising clinical practice of HER2-low cancer, as well as other advanced breast cancer.

TNBC, with aggressive behaviour and poor prognosis, remains the most challenging type of breast cancer to treat. Chemotherapy is the standard of care for TNBC besides surgery, and targeted therapies such as poly (ADP-ribose) polymerase inhibitors, immune checkpoint inhibitors, and ADCs have shown benefits to patients. According to the Keynote-522 data published in 2023, addition of the anti-programmed cell death protein-1 antibody pembrolizumab to neoadjuvant chemotherapy significantly improved the percentage of patients with a pathological complete response. However, TNBC is highly diverse with various histopathological landscapes, leading to off-target and drug resistance.

Along with numerous exploratory research and breakthroughs in subtype-based therapies, omics technologies are refining our understanding of breast cancer subtypes beyond classic histological features, multigene expression, and immune profiling. Recent multiomics profiling of breast cancer in the Chinese population put forward further molecular classifications of TNBC, HR-positive, and HER2-low breast cancers. These data enable us to consider the unique characteristics of each patient's cancer rather than treating all patients with the same therapy. For example, based on the four transcriptome-based subtypes of TNBC, a series of FUTURE trials assessing the efficacy of subtyping-based regimens are now underway. Intriguingly, data of the FUTURE-SUPER trial published in January, 2024, showed a 5.5 months longer PFS in the subtyping-based group than in the control group, highlighting the encouraging potential clinical benefits of using molecular subtype-based treatment optimisation in patients with TNBC.

Despite notable advances in precision treatment, several challenges remain in achieving favourable outcomes for patients. First, population-based genotyping should consider the heterogeneity among different nations and ethnicities, which largely limits generalisability. Variations can also exist among diagnosing and treatment sites due to the absence of standardised genotyping and treatment approaches. Second, the cost of genotyping and personalised treatment will be high. Multiomic machine learning models to predict the intrinsic subtypes and response to treatments could be a solution, but they must rely on large scale datasets and robust validations. Finally, there is still a long way to go from subtyping to targeted precision therapies. Future research will not only require the development of individualised strategies based on existing treatments, but even more the exploration of new drugs specific to novel subtypes. Currently there has been limited availability of clinical trials for precision treatments despite the increasing knowledge of molecular subtypes, making it difficult to determine their true value in treatment. For example, the ongoing FUTURE trials focus on Chinese population in large medical centre receiving disproportionately large amount of resources, might not be representative of the rest of the Western Pacific region population and clinical scenario. Trials conducted in the Western Pacific region that contextualise the state of precision medicine in the regional population are needed.

*The Lancet Regional Health – Western Pacific* welcomes and calls for medical studies exploring more specific actionable biomarkers, real-word data assessing efficacy of precision therapies, and research regarding affordability and standardisation in molecular testing and treatment approaches. Eventually, we hope to see revolutions in precision therapies for breast cancer to ensure the optimisation of treatment for every patient.

